# Non steroidal anti-inflammatory drugs-induced fixed drug eruptions: a case series

**DOI:** 10.1186/2045-7022-4-S3-P82

**Published:** 2014-07-18

**Authors:** Karim Aouam, Najah Ben Fadhel, Nadia Ben Fredj, Amel Chaabane, Hichem Belhadjali, Naceur A Boughattas, Karim Aouam

**Affiliations:** 1 faculty of medecine of Monastir, departement of pharmacology, Tunisia

## Introduction

Fixed drug eruption (FDE) is a pattern of a drug-induced skin reaction. It is characterized by skin erythematous plaques that recur at the same site each time the drug is administered. Several drugs have been associated to such cutaneous reaction, including, anticonvulsant agents, sulfonamides, non opioid analgesics and tetracyclines. Non steroidal anti-inflammatory drugs (NSAIDs) have been also implicated in inducing FDE.

## Aims

To report a case series of NSAIDs-induced FDE confirmed by a positive patch test and/or a positive rechallenge with the suspected drug and to assess the cross reactivity between NSAIDs.

## Materials and methods

We included all cases of NSAIDs-induced FDE, using the side effects database of the unit of pharmacovigilance of Monastir. The drug imputability of FDE was established according to Naranjo method. Patch tests were performed in the involved and normal skin following the ENDA recommendations.

## Results

Among 22 cases of FDE, recorded in our database, eight (36%) have been considered to be related to NSAIDs. There were 6 male and 2 female patients. The mean age of our patients was 44 years old. Six out of eight patients have a multiple fixed drug eruption and two among them have bullous eruption. The time between drug intake and skin symptoms averaged two days. Suspected NSAIDs were mefenamic acid (n=5), piroxicam (n=2) and acetyl salicylic acid (n=1). Patch tests were performed for seven patients. In one case, the patch test was negative, however the rechallenge of mefenamic acid was positive. All the remaining tests were positive, mefenamic acid (n=3), piroxicam (n=2) and salicylic acid (n=1). Rechallenge of the culprit drug was done for five patients and there were all positive. Based on the Naranjo algorithm, it is definite that FDE was due to NSAIDs in seven cases. Cross reactivity was assessed in six patients (using patch tests and/or a provocation test of other chemically different NSAIDs) and was negative in all of them.

## Conclusion

We add to the medical literature eight cases of FDE induced by NSAIDs. Mefenamic acid was the most involved drug in our serie. NSAIDs induced FDE seems to be selective as far as we did not show a cross reactivity between these drugs.

